# The Effect of Adhesive Systems on the Bond Strength of Directly Bonded Fixed Partial Dentures Using Artificial Teeth: An In Vitro Study

**DOI:** 10.7759/cureus.93910

**Published:** 2025-10-06

**Authors:** Adityakrisna Yoshi Putra Wigianto, Yuichi Ishida, Megumi Watanabe, Tetsuo Ichikawa

**Affiliations:** 1 Department of Prosthodontics and Oral Rehabilitation, Tokushima University Graduate School of Biomedical Sciences, Tokushima, JPN

**Keywords:** bond strength, denture artificial tooth, direct bonded fixed partial denture, fixed partial denture, resin cement

## Abstract

Objective

This study aimed to evaluate the effect of different adhesive systems on the bond strength of a directly bonded fixed partial denture (DBFPD) utilizing artificial teeth through shear bond strength (SBS) testing and a DBFPD model experiment.

Materials and methods

Two different adhesive systems: self-curing resin cement (SRC) (Super Bond Universal; Sun Medical, Moriyama, Japan) and light-curing flowable resin composite (LFRC) (G-Fix; GC, Tokyo, Japan) were applied to two different hybrid resin artificial teeth: Duracross Physio (Nissin, Kyoto, Japan) and Endura Posterio (Shofu, Kyoto, Japan). The SBS of the adhesive systems to artificial teeth was evaluated using a universal testing machine (AGX-1kN, Shimadzu, Kyoto, Japan). Subsequently, the failure modes were assessed using a stereomicroscope (VHX-970F, Keyence, Osaka, Japan). An experimental model comprising a lower mandibular incisor artificial tooth as a pontic was utilized to evaluate the bond strength of these adhesive systems, with or without a retentive groove.

Results

The SRC group (≈20 MPa) exhibited significantly higher SBS than the LFRC group (≈10MPa), with no significant difference between artificial teeth types. Failure mode analysis showed no adhesive failure in the SRC group, while the LFRC group primarily failed adhesively. In the model experiment, SRC-fixed artificial teeth had the highest bond strength, unaffected by groove presence. The LFRC group with a groove showed slightly improved strength, but the increase was not statistically significant.

Conclusions

SRC demonstrated superior bond strength compared to LFRC for hybrid resin artificial teeth, regardless of filler percentage or retentive groove presence.

## Introduction

The resin-bonded fixed partial denture (RBFPD) has been widely used in prosthodontic treatment thanks to the sustained development of adhesive materials. In contrast to the conventional fixed partial denture (CFPD), which requires extensive abutment tooth preparation, RBFPD can be fixed with minimal enamel reduction or even without any reduction. Consequently, it is advantageous, especially in preserving the integrity of adjacent tooth surfaces. Reported survival rates range from 84.8 to 95.01% after five years, depending on the materials and number of retainers [1].

Although the RBFPD is more cost-effective than CFPDs or fixed implant prostheses, this indirect technique still requires laboratory work to manufacture using materials such as ceramics, metals, or fiber-reinforced composite [2]. As an alternative, a directly bonded fixed partial denture (DBFPD) technique has been introduced, offering a simplified procedure that can be completed entirely chairside without the need for impressions or additional laboratory steps [3]. Initially, this method was used as a temporary prosthodontic method, where an artificial tooth or extracted natural tooth was bonded to the pontic portion with an adhesive material. Consequently, the DBFPD is particularly beneficial for cases requiring same-day treatment or for dentists working in settings with limited facilities, such as rural areas without access to dental laboratories or in countries where health insurance does not cover fixed dental prostheses (FDPs).

Clinical studies on the use of DBFPD are limited. Previously reported pontic options include an extracted natural tooth [4], a composite resin artificial tooth [5], or an acrylic resin artificial tooth [6]. Methyl methacrylate-based resin and composite resin luting agents have been used to fix DBFPD to adjacent teeth [3]. The success of DBFPD treatment is dependent on several key factors, including the appropriate selection of pontic material, the surface properties of the bonding areas, the choice of adhesive, and the bonding procedure itself. However, in vitro studies evaluating the bond strength of artificial teeth primarily focus on analyzing their adhesion to the denture base [7].

The 4-methacryloyloxylethyl trimellitate anhydride (4-META)/methyl methacrylate (MMA)-tri-n-butyl borane (TBB) resin luting agent has been frequently used for distal base-free posterior fixed dental prostheses (DBFPD) and the splinting of mobile teeth, demonstrating satisfactory bond strength and clinical outcomes. Recently, a Bis-EMA and phosphoric ester monomer-based flowable resin paste has been evaluated in a study as a potential resin-based material for posterior FDPs [8]. Although clinical applications of DBFPDs using artificial teeth have been reported in a few studies [5,6,9], the optimal conditions for bonding artificial teeth to adjacent natural teeth remain unclear. Factors such as artificial tooth type and composition, surface treatment, and adhesive material selection require further investigation.

Previous research has explored the use of both 4-META/MMA-TBB resin and Bis-EMA-based resin in adhesive dentistry. However, a systematic literature search conducted to identify previous studies comparing the bond strength of these two adhesive materials for DBFPDs with artificial teeth returned no relevant results. Therefore, this study aimed to evaluate the bond strength and failure modes of two adhesive systems applied to different artificial teeth compositions and the presence of a retentive groove to determine an effective adhesive system for DBFPD.

## Materials and methods

Materials

Two types of hybrid composite resin artificial teeth were used in this study: Duracross Physio (Nissin Dental Products; Kyoto, Japan) and Endura (Shofu, Kyoto, Japan). Two different adhesive resin systems were utilized: a self-cure resin cement, Super Bond Universal (Sun Medical, Moriyama, Japan), and a light-cure flowable resin composite, G-Fix (GC, Tokyo, Japan), hereinafter abbreviated as SRC and LFRC, respectively. The chemical surface treatments applied were M&C Primer (Sun Medical, Moriyama, Japan) and GC Ceramic Primer II (GC; Tokyo, Japan). The detailed compositions of these materials are provided in Table 1, while the specific application procedures, based on the manufacturers' instructions, are outlined in Table 2.

**Table 1:** Materials used in this study

Material type	Identification	Composition		Manufacturer
Artificial tooth	Duracross	Composite resin (68% filler content)		Nissin Dental Products, Kyoto, Japan
	Endura	Composite resin (47% filler content)		Shofu, Kyoto, Japan
Surface treatment	M&C Primer	Primer A: MMA, MDP, VBATDT, Acetone Primer B: MMA, γ-MPTS		Sun Medical, Moriyama, Japan
	GC Ceramic Primer-II	γ-MPTS (γ-methacryloxypropyl trimethoxysilane), 10-MDP (10-methacryloyloxydecyl dihydrogen phosphate), MDTP (methacryloyloxydecyl dihydrogen thiophosphate), BisGMA (bisphenol A-glycidyl methacrylate), TEGDMA (triethylene glycol dimethacrylate), ethanol		GC, Tokyo, Japan
Fixation material	Self-cure resin cement (SRC): Super Bond Universal	Quick monomer: methyl methacrylate (MMA), 4-methacryloxyethyl trimellitate anhydride (4-META). Catalyst V: tri-n-butylborane (TBB). Powder: polymethyl methacrylate (PMMA)		Sun Medical, Moriyama, Japan
	Light-cure flowable resin composite (LFRC): G-Fix	Resin paste: barium glass silane-treated filler (26%), methacrylate ester, phosphate ester monomers, photoinitiator		GC, Tokyo, Japan

**Table 2:** Application procedures of surface treatments and fixation materials

Material	Application procedure			
M&C Primer	Dispense equal drops of primer A and primer B into a disposable mixing well, mix with a microbrush, then apply to a clean adhesion surface			
Super Bond Universal (Brush-dip technique)	Prepare a polymer powder in the dispensing dish, flatten the surface, and prepare the activated liquid by mixing an appropriate amount of quick monomers and catalyst V with a 4:1 drops ratio. Afterward, wet a brush tip with the activated liquid, then touch the polymer powder with the brush tip, forming a small polymer ball. Finally, transfer and apply the polymer balls onto the isolated bonding surface adequately			
GC Ceramic Primer-II	Dispense one drop of ceramic primer II into a dispensing dish, then apply as a thin layer to a clean adhesion surface using a microbrush			
G-Fix	Dispense the flowable resin paste onto the adhesion surface. Light cure for 10 seconds			

Shear bond strength (SBS)

**Figure 1:**
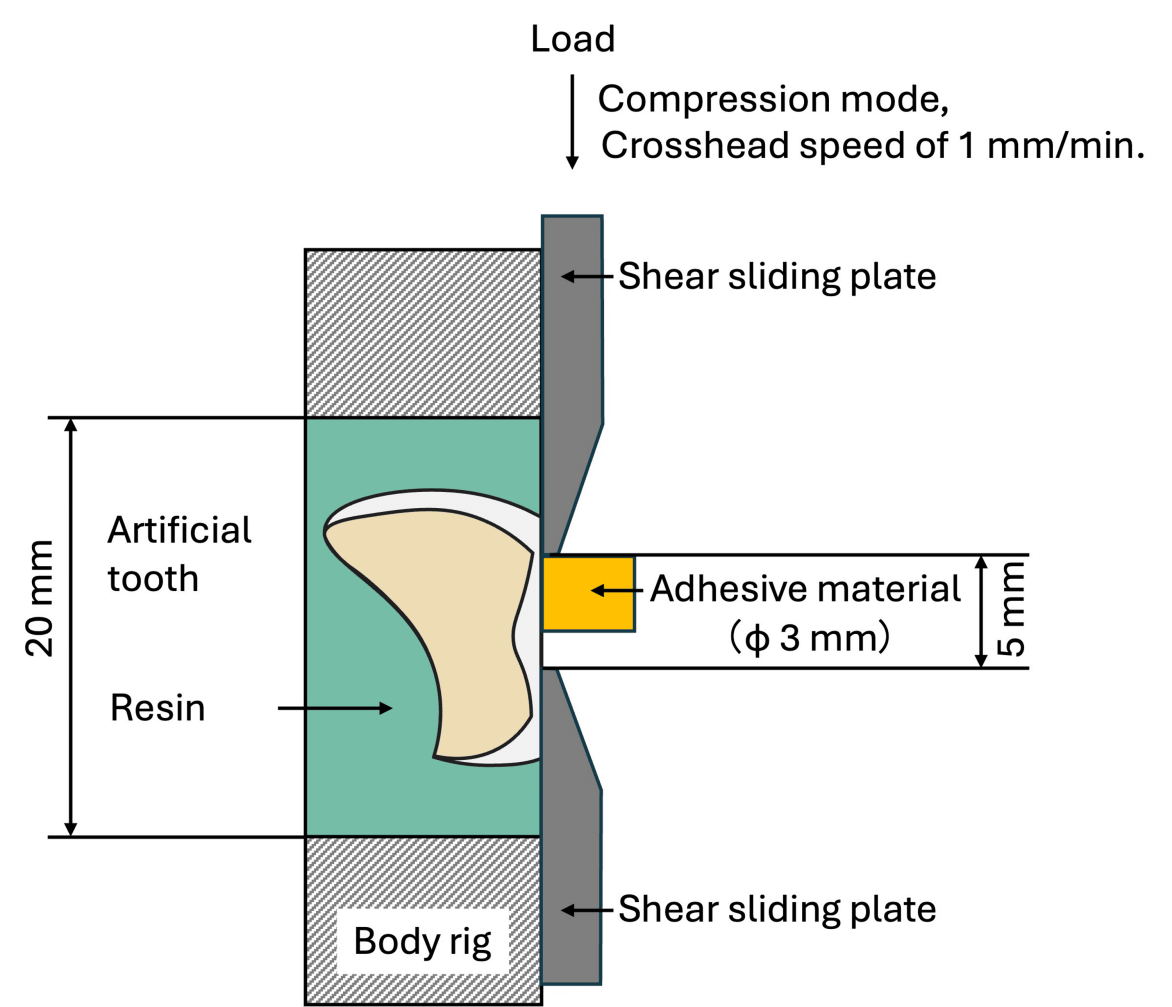
Illustration of shear bond strength evaluation test performed in this study

The SBS of the artificial tooth enamel equivalent and the adhesive material was evaluated. The in vitro experiment setting is shown in Figure 1. The substrate in this study was prepared by initially flattening the artificial teeth surface with a carborundum bur (Shofu; Kyoto, Japan) using a micromotor, then embedding it in a specimen embedding resin - Technovit 4071 (Kulzer, Hanau, Germany) - by using a cylindrical mold that fits the size of the shear bond strength (SBS) testing jig. Subsequently, the embedded artificial tooth underwent polishing with a #600-grit silicon carbide paper using a specimen preparation polishing machine to ensure a flush surface.

Before the adhesion procedures, the surfaces of embedded artificial teeth were sandblasted with 50 μm aluminium oxide at 2 MPa, cleaned in an ultrasonic cleaner with ethanol, and thoroughly dried. Afterwards, the adhesion area was isolated into a 3 mm diameter using a tape with a 3 mm punched hole. The chemical surface treatments were then applied as per the manufacturer’s instructions on the isolated area. A mold was then placed on the isolated surface, and then the adhesive materials were prepared and applied onto the substrate inside the mold. The SRC was applied by applying resin balls made from picking the polymer using a brush dipped in their activated liquid, then chemically polymerized after six minutes. On the other hand, the LFRC system was applied by dispensing the resin in the form of a flowable resin paste continuously, then polymerized using a visible blue LED light with 2000 mW/cm^2^ at 460 nm wavelength Pencure 2000 (Morita, Kyoto, Japan) for 10 seconds. Subsequently, the samples were stored in distilled water for 24 hours at 37 °C.

The SBS tests were performed using an SBS testing jig installed on a universal testing machine AGX-1kN (Shimadzu, Kyoto, Japan) with a crosshead speed of 1 mm/min. The surface of debonded samples following the SBS test was evaluated using a digital stereomicroscope (VHX-970F, Keyence, Osaka, Japan) to assess the failure types into the following three categories: adhesive: fracture occurred at the interface between the adhesive material and the artificial tooth; cohesive: occurred within the artificial tooth without any adhesive material failure; and mixed: occurred on both artificial tooth and adhesive material. Fifteen specimens were prepared in each condition.

Model experiment

**Figure 2:**
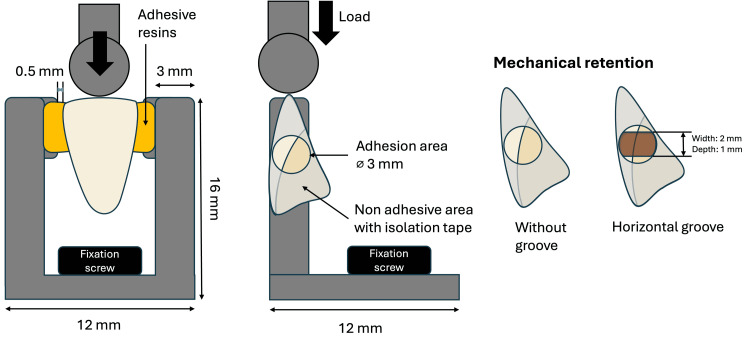
Illustration of the model experiment configurations performed in this study and the artificial tooth mechanical retention structure explanation

The model experiment samples were prepared using lower incisor Duracross Physio as a pontic in two groups, based on the adhesive materials (SRC and LFRC). Each group was further subdivided into two based on the presence and absence of a retentive groove (a 2 mm-wide, 1 mm-deep horizontal groove was made on the proximal surface using a ⌀2 mm carbide bur) (Figure 2). In total, there were four groups evaluated (SRC without groove; SRC with groove; LFRC without groove; LFRC with groove). Subsequently, the proximal surfaces were sandblasted, cleaned with ethanol using an ultrasonic machine, and then isolated using tape as described in the SBS test to control the adhesion area. Custom metal jigs made of cobalt chromium were fabricated, referring to a method reported by Nakajima et al. [10].

To ensure an identical position of the pontic toward the metal jig, a silicone rubber, Labocone putty (GC; Tokyo, Japan) was utilized as the pontic holder in all samples during the adhesive procedures until complete polymerization. Afterwards, the adhesive materials were applied to adhere the pontic to the jig as per the manufacturer's instructions. Following 24-hour immersion in distilled water, the samples were fixed onto the universal testing machine by using a bolt screw, then an axial load was applied to the center of the incisal surface using a compression mode with a crosshead speed of 1 mm/min (Figure 2). The maximum load required to detach the artificial tooth from the model was recorded as a failure load in Newton (N), along with the failure locations: metal-jig or artificial tooth side. Five specimens were prepared in each condition.

Statistical analysis

The Shapiro-Wilk test was conducted to verify the normality of the collected datasets, and then parametric statistical analysis was carried out. One-way analysis of variance (ANOVA) with Tukey’s post-hoc was performed to investigate the significant differences between groups in SBS evaluation. The Kruskal-Wallis test was conducted with pairwise comparisons to investigate the significant differences between groups in model bond strength evaluations. Afterwards, a Bonferroni correction was performed to adjust the p-values. The significance level was set at 0.05, and the statistical analysis software IBM SPSS Statistics version 25 (IBM Corp., Armonk, NY) was utilized.

## Results

SBS results

**Figure 3:**
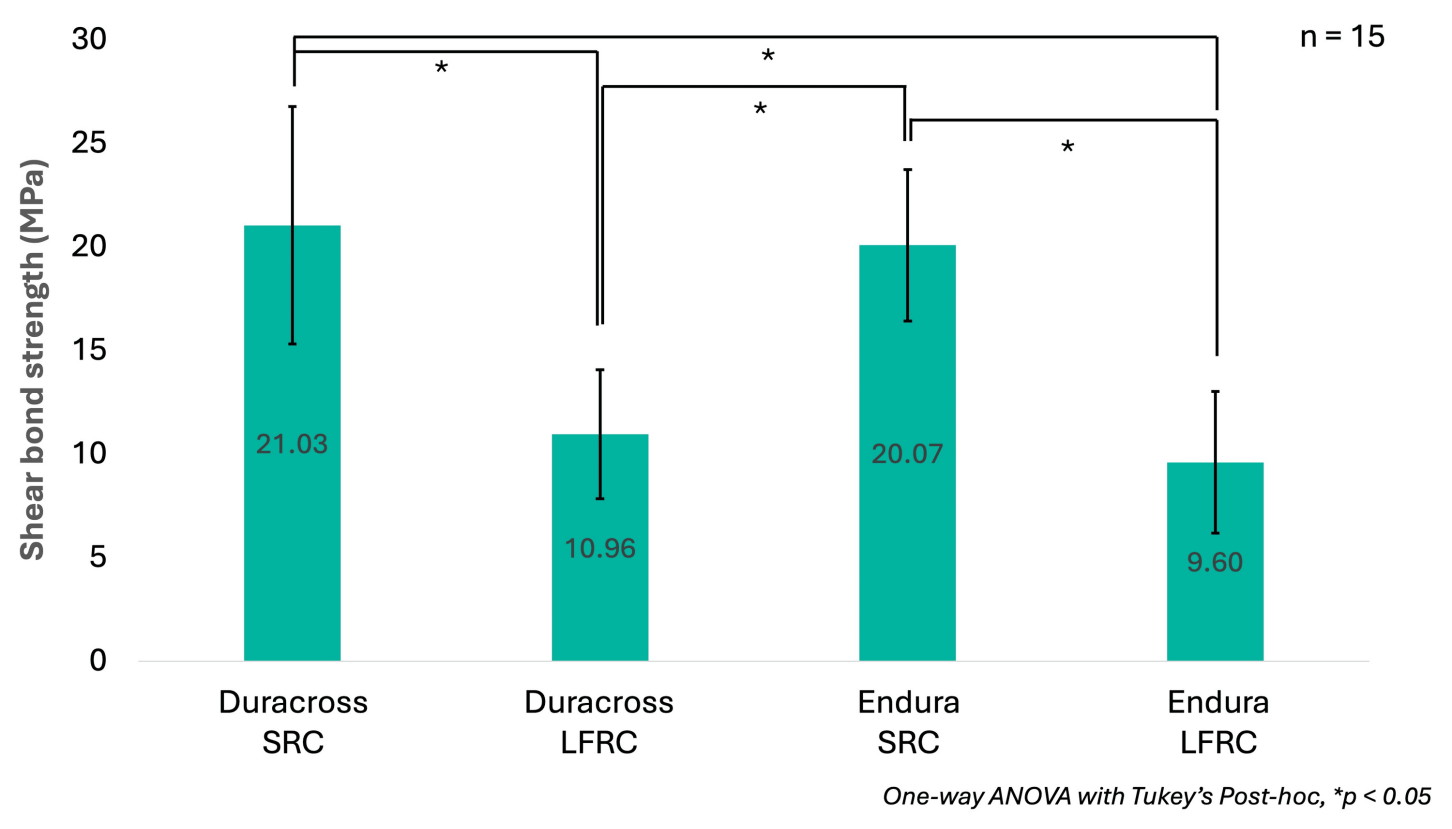
Shear bond strength comparison between different artificial tooth and fixation materials Numbers inside the bars represent the mean, while the T-bars indicate the standard deviation SRC: self-curing resin cement; LFRC: light-curing flowable resin composite

Figure 3 shows the means and standard deviations (SD) of SBS in each condition. The highest SBS was found on the Duracross SRC group (21.03 ± 5.73 MPa), followed by Endura SRC (20.07 ± 3.65 MPa), Duracross LFRC (10.96 ± 3.11 MPa), and Endura LFRC (9.6 ± 3.42 MPa), respectively. The One-way ANOVA result on SBS evaluation revealed that there was a statistically significant difference in SBS between groups (p = 0.000). Based on Tukey’s post-hoc results, the significant SBS differences were only evident between the different adhesive materials (p = 0.000), whereas no significance was observed based on different artificial teeth (p = 0.967 within Duracross SRC and Endura SRC; p = 0.841 within Duracross LFRC and Endura LFRC).

Failure modes

**Figure 4:**
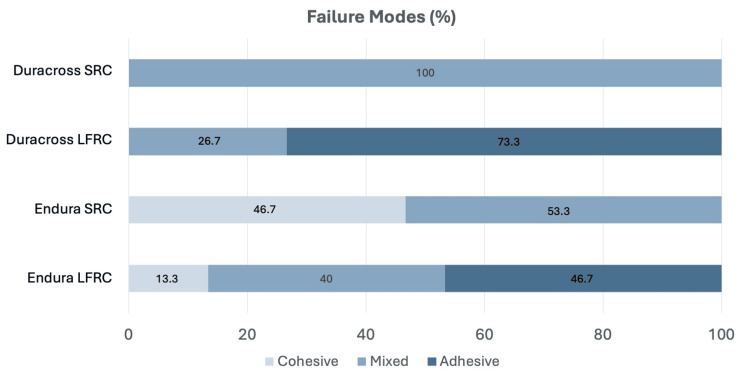
Failure mode distribution percentage in all groups following shear bond strength evaluation SRC: self-curing resin cement; LFRC: light-curing flowable resin composite

**Figure 5:**
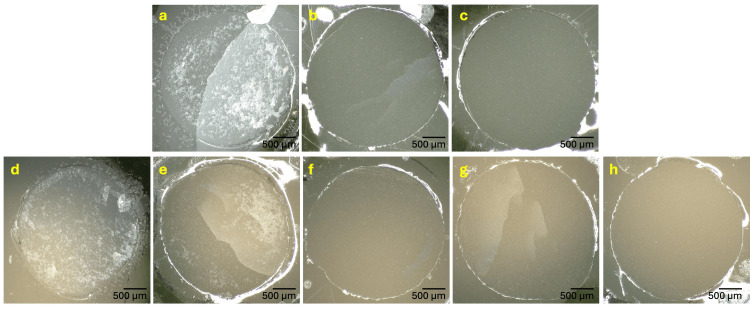
Representative stereomicroscopic images at 50× magnification of each failure modes Upper panel (Duracross): a. SRC mixed, b. LFRC mixed, and c. LFRC adhesive. Lower panel (Endura): d. SRC cohesive, e. SRC mixed, f. LFRC cohesive, g. LFRC mixed, and h. LFRC adhesive SRC: self-curing resin cement; LFRC: light-curing flowable resin composite

The failure mode distributions from all groups after SBS evaluation are provided in Figure 4, with representative stereomicroscopic images provided in Figure 5. When fixing an artificial tooth with SRC, no adhesive failure was observed. In the Duracross SC group, mixed failures were observed in all samples (100%), whereas 53.3% mixed failures and 46.5% cohesive failures on SRC were seen on the Endura artificial tooth. In contrast, adhesive failures were observed in the LFRC groups. The adhesive failure of LFRC-fixed Duracross was 73.3%, followed by 26.7% of mixed failures. Similarly, failure modes of LFRC-fixed Endura were dominantly adhesive (46.7%), followed by mixed (40%), and cohesive on LFRC (13.3%), respectively.

Model experiment

**Figure 6:**
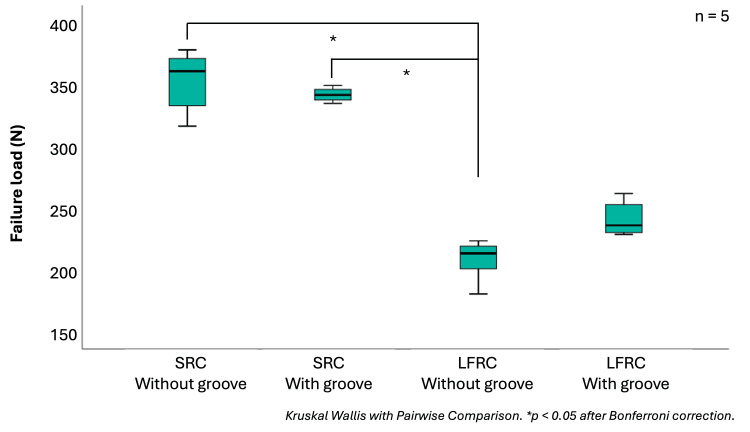
Box plots of failure loads measured in the model experiment representing median, upper quartile, lower quartile, maximum, and minimum value of each dataset SRC: self-curing resin cement; LFRC: light-curing flowable resin composite

Figure 6 shows the box plots of the model experiment results, and the descriptive data are provided in Table 3. SRC-fixed artificial tooth without groove demonstrated the highest bond strength (345 ± 26 N), followed by SRC with groove (342 ± 16 N), LFRC with groove (237 ± 14 N), and LFRC without groove (204 ± 17 N). Similar to the SBS results, the bond strengths of SRC without and with groove were significantly higher than those of the LFRC without groove (p = 0.005, p = 0.006, respectively). The presence of a groove in the LFRC group resulted in a slightly higher bond strength, but it was not statistically significant (p = 1.000). Moreover, the bond strength of the LFRC with groove also did not significantly differ from either SRC without groove (p = 0.253) or SRC with groove (p = 0.288). Regarding failure locations, all LFRC without groove models failed on the artificial tooth surface, whereas LFRC with groove demonstrated 60% tooth and 40% metal side. The SRC without a groove failed more likely in the metal side (60%) than the tooth side (40%), whereas the groove presence demonstrated more tooth side failure (80%) than metal (20%).

**Table 3:** Model experiment results Different superscript characters (^a^, ^b^, ^ab^) indicate a statistically significant difference between groups (p<0.05). Groups sharing a common superscript letter (e.g., ^a^ and ^ab^) are not significantly different from each other SD: standard deviation; SRC: self-curing resin cement; LFRC: light-curing flowable resin composite

Groups	Mean ± SD (N)	Median	Lower quartile	Upper quartile
SRC without groove	345 ± 26^a^	354	338	367
SRC with groove	342 ± 16^a^	336	331	346
LFRC without groove	203 ± 17^b^	209	187	217
LFRC with groove	237 ± 14^ab^	232	225	252

## Discussion

In recent years, treatment methods that minimize invasiveness and are completed chairside as much as possible have been promoted. There are various options in providing fixed partial dentures directly, such as fiber-reinforced [11], metal wire-reinforced composites in which the pontics are built manually [12], injecting flowable composite into a custom mold technique [13], and utilizing an artificial tooth as a pontic in DBFPD. DBFPD utilizing artificial tooth provides several advantages, such as minimum effort, unlike manually sculpting pontic morphology, which relies on individual skills, thus saving time and number of visits. DBFPD is considered a simple method that makes it easier to ensure both aesthetic and functional qualities. Artificial teeth are also very stable in terms of materials science.

The DBFPD technique is indicated for cases where the pontic space between abutment teeth is narrow, such as in a mandibular anterior tooth. However, in cases like when highly characterized morphologies or tooth shades are desired and large interproximal spaces exist, this method may not address those demands appropriately, as the shape and shades of the artificial teeth are prefabricated. Although factors such as occlusal equilibration, patient cooperation, and adequate posterior occlusal support should be carefully considered [3], particular attention must also be given to the bond strength and the condition at the fixation sites, as these are critical aspects that warrant thorough investigation. The two adhesive materials used in this study have been previously reported to bond with natural teeth favorably. The SBS of SRC to enamel with prior surface acid-etching was reported to be around 30 MPa [14,15], whereas the LFRC was reported at 24 MPa [16]. Both materials are considered potential for DBFPD with artificial tooth owing to their simple application procedures, but differ in application and polymerization method. On the other hand, there have not been enough reports on their adhesion to artificial teeth.

The present study suggested that the SBS of both adhesives to artificial teeth evaluated in this study was lower than their reported SBS to enamel. Therefore, DBFPD with artificial teeth utilizing these adhesives would be more likely to fail on the hybrid-resin artificial tooth side than the abutment side, which is clinically desirable as it leaves the abutment tooth undamaged [17]. Currently, there is no consensus on a clinically acceptable SBS range for FPDs. Previous studies reported a wide range of SBS as it varies with adhesive materials and procedures, substrates, and adherends evaluated. For example, the SBS of various ceramic RBFPDs to enamel ranges from 6 to 31 MPa [18]. Another study reported the SBS of fiber-reinforced composite resin FPDs within 12-21 MPa [19]. The SBS values of 10-20 MPa indicated in this study are speculated to be a clinically acceptable range.

The higher SBS of SRC groups than that of LFRC groups corroborates previous studies, reporting that MMA-based resin cements exhibited stronger bond strength than composite resins due to their ability to penetrate polymer chain interspaces, forming a semi-interpenetrating polymer network [20]. The SRC used in this study is a self-curing resin initiated by TBB, resulting in a long polymerization time, allowing longer infiltration on the artificial tooth surface [21]. In contrast, LFRC relies on a photo/light-initiated polymerization process, increasing the risk of polymerization shrinkage stress on the adhesive interface, reducing the bond strength [22]. Moreover, G-Fix has higher flexural strength (239 MPa) and modulus of elasticity (25 GPa) than Super Bond (159 MPa; 16 GPa, respectively) [23,24]. These mechanical properties have been reported to affect SBS [25], which may explain the findings of this study. As addressed by Tanoue et al. [3], despite its lower mechanical properties, plastic deformations in MMA-based resins may be advantageous in DBFPD applications. Within the same adhesive system applied to different artificial teeth, there was no significant difference in the SBS. This result might indicate that the filler content difference between Duracross Physio (68%) and Endura Posterio (47%)’s outermost hybrid resin layer does not affect SBS, which aligns with the study by Han et al. (2020) [26].

Further investigation on the failure mode revealed that no adhesive failure was observed in the SRC groups, whereas most of the LFRC groups failed adhesively. Adhesive failures observed in LFRC groups may be attributed to weak bonding at the adhesion interface. Despite no SBS difference, the failure mode characteristics differed between Duracross and Endura. When adhered with SRC, mixed failure was evident on all debonded Duracross samples, whereas both mixed and cohesive failure of SRC were observed on Endura. Similarly, Han et al. (2020) also reported more cohesive failures of resin cement in Endura, without a significant difference in SBS [26]. The lower filler percentage of Endura might allow for greater penetration of adhesive resins into the matrix, resulting in these findings.

The bond strength of the DBFPD was examined in a more clinically relevant model experiment. The model experiment results (204-345 N) suggest that the adhesive systems tested in this study exhibited potential load resistance that exceeds the average reported functional forces loaded to the anterior region (108 to 185 N) [27,28]. It is also comparable to previous anterior RBFPD reports, which vary widely from 210 to 724 N [29,30], which mostly employed solid connector structures, whereas the connector in this study was merely adhesive resins. Although not statistically significant, the presence of a horizontal groove slightly reduced the bond strength in SRC, whereas it increased the bond strength in the LFRC group. Therefore, the presence of an additional retentive groove on an artificial tooth may be beneficial when fabricating DBFPDs using composite resin, but not with the MMA-based resin cements.

Similarly, a study by Nakajima et al. reported that the presence of holes, grooves, rest, and pins did not significantly affect the bond strength of 4-META/MMA-TBB compared to a silane-treated artificial tooth without mechanical reinforcement [10]. Clinicians should meticulously consider material selection to achieve successful treatment. Composite materials with higher mechanical properties may be more suitable when utilized as the main component of composite FPDs, as it was reported to perform well in an in vitro evaluation for a fiber-reinforced molar inlay retained FPD [8]. Based on the results from this in-vitro study, the characteristics of MMA-based resins appear more effective for achieving bond integrity on hard resin artificial teeth, as they provided consistently higher bond strength values and more favorable failure modes than the LFRC.

There are some limitations in this in vitro study that need to be considered when interpreting the results. As an in vitro investigation, this study is inherently limited by the challenges of fully simulating the dynamic and complex oral environment. The bond strength and failure load were measured after 24-hour immersion in distilled water, only representing a short-term condition without the durability aspects simulating more extensive clinical conditions, such as cyclic loading and thermal cycling. In the model experiment, a metal jig was utilized as abutment teeth instead of natural extracted incisors to minimize uncontrollable anatomical variations of extracted natural teeth; thus, the estimated failure load values might differ when bonded to enamel clinically. The scope of this study was limited by the small number of specific adhesive systems and artificial tooth compositions tested, which may restrict the broad generalizability of our findings. Further in vitro and rigorous clinical studies on DBFPD utilizing artificial teeth should be conducted to clarify these aspects.

## Conclusions

The present in vitro study highlights the impact of the adhesive material selection on DBFPD using an artificial tooth as a pontic. The 4-META/MMA-TBB self-curing resin cement demonstrated better bond strength to hybrid resin artificial teeth than the light-curing flowable resin composite for tooth splinting material. This better performance was observed under static laboratory testing conditions, irrespective of the differences in artificial tooth filler percentages or the presence of a retentive groove. These results should be interpreted with caution when extrapolating to long-term clinical outcomes, which are subject to factors such as dynamic fatigue, thermal changes, and material degradation.

## References

[1] Alraheam IA, Ngoc CN, Wiesen CA, Donovan TE (2019). Five-year success rate of resin-bonded fixed partial dentures: a systematic review. J Esthet Restor Dent.

[2] Mendes JM, Bentata AL, de Sá J, Silva AS (2021). Survival rates of anterior-region resin-bonded fixed dental prostheses: an integrative review. Eur J Dent.

[3] Tanoue N, Takeuchi Y, Furuchi M, Yamamori T, Ohkawa S (2021). Direct bonded fixed partial denture with an artificial denture tooth as a pontic. Jpn Dent Sci Rev.

[4] Nakamura M, Nogawa H, Matsumura H (2015). Eleven-year clinical performance of a mandibular natural tooth pontic bonded with modified tri-n-butylborane initiated adhesive resin. J Oral Sci.

[5] Tanoue N, Tanaka T (2015). A direct bonded fixed partial dental prosthesis: a clinical report. J Prosthet Dent.

[6] Monya Y, Matsumura H, Atsuta M (1998). A two-stage resin-bonded fixed partial denture seated in conjunction with postextraction healing of the alveolar socket: a clinical report. J Prosthet Dent.

[7] Prpic V, Catic A, Kraljevic Simunkovic S, Bergman L, Cimic S (2023). The shear bond strength between milled denture base materials and artificial teeth: a systematic review. Dent J (Basel).

[8] Cekic-Nagas I, Egilmez F, Ergun G, Vallittu PK, Lassila LV (2018). Load-bearing capacity of novel resin-based fixed dental prosthesis materials. Dent Mater J.

[9] Shimizu H, Kawaguchi A, Moriguchi S, Tanaka T, Atsuta M (1986). Study on the provisional fixed prosthodontic treatments by the use of acrylic resin teeth. Nihon Hotetsu Shika Gakkai Zasshi.

[10] Nakajima Y, Minesaki Y, Miyazato A (1999). Study on adhesion fixed partial denture applied composite resin teeth as pontic. In vitro measurement of bonding durability. Nihon Hotetsu Shika Gakkai Zasshi.

[11] Perrin P, Meyer-Lueckel H, Wierichs RJ (2020). Longevity of immediate rehabilitation with direct fiber reinforced composite fixed partial dentures after up to 9 years. J Dent.

[12] Wierichs RJ, Weilenmann W, Jeganathan S, Perrin P (2022). Longevity of immediate rehabilitation with direct metal-wire reinforced composite fixed partial dentures. Dent Mater.

[13] Hosaka K, Tichy A, Hasegawa Y, Motoyama Y, Kanazawa M, Tagami J, Nakajima M (2021). Replacing mandibular central incisors with a direct resin-bonded fixed dental prosthesis by using a bilayering composite resin injection technique with a digital workflow: a dental technique. J Prosthet Dent.

[14] Nogawa H, Koizumi H, Saiki O, Hiraba H, Nakamura M, Matsumura H (2015). Effect of a self-etching primer and phosphoric acid etching on the bond strength of 4-META/MMA-TBB resin to human enamel. Dent Mater J.

[15] Kodaira A, Koizumi H, Nogawa H, Okamura K, Nakamura M, Yoneyama T (2019). Effect of a self-etching primer containing 4-META and sodium sulfite after phosphoric acid etching on bonding strength of MMA-TBB resin to human enamel. J Prosthodont Res.

[16] Lee YR, Kim SY, Kim JW, Park SH, Cho KM (2019). Comparison of adhesive strength of resinous teeth splinting materials according to enamel surface treatment. J Dent Rehabil Appl Sci.

[17] Nujella BP, Choudary MT, Reddy SP, Kumar MK, Gopal T (2012). Comparison of shear bond strength of aesthetic restorative materials. Contemp Clin Dent.

[18] Roman T, Cournault B, Teyagirwa PF (2024). Shear bond strength between standard or modified zirconia surfaces and two resin cements incorporating or not 10-MDP in their matrix. Dent Mater.

[19] Antonopoulou A, Papadopoulos T, Hatzikyriakos A (2012). In vitro evaluation of shear bond strength and mode of failure of the interface between an indirect composite bonded to fiber-reinforced composite substructures. J Prosthodont.

[20] Hata K, Komagata Y, Nagamatsu Y, Masaki C, Hosokawa R, Ikeda H (2023). Bond strength of sandblasted PEEK with dental methyl methacrylate-based cement or composite-based resin cement. Polymers (Basel).

[21] Hirabayashi C, Imai Y (2002). Studies on MMA-tBB resin. I. Comparison of TBB and other initiators in the polymerization of PMMA/MMA resin. Dent Mater J.

[22] Ferracane JL, Hilton TJ (2016). Polymerization stress--is it clinically meaningful?. Dent Mater.

[23] Yoo JI, Kim SY, Batbayar B, Kim JW, Park SH, Cho KM (2016). Comparison of flexural strength and modulus of elasticity in several resinous teeth splinting materials. J Dent Rehabil Appl Sci.

[24] Moon W, Hyun HK, Chung SH (2022). Mechanical evaluation of dental trauma splints fabricated using recently-developed photo-polymerizable composites. Dent Mater J.

[25] Irie M, Okada M, Maruo Y, Nishigawa G, Matsumoto T (2023). Shear bond strength of resin luting materials to lithium disilicate ceramic: correlation between flexural strength and modulus of elasticity. Polymers (Basel).

[26] Han SY, Moon YH, Lee J (2020). Shear bond strength between CAD/CAM denture base resin and denture artificial teeth when bonded with resin cement. J Adv Prosthodont.

[27] Singh A, Tandon P, Singh GK, Nagar A, Shastri D (2022). Maximum voluntary molar and incisor biting force and morphological variables in subjects with different vertical skeletal patterns. J Indian Orthod Soc.

[28] Al-Wahadni A, Dkmak MS, Almohammed S, Hatamleh MM, Tabanjah A (2024). Fracture strength of anterior cantilever resin-bonded fixed partial dentures fabricated from high translucency zirconia with different intaglio surface treatments. J Prosthodont.

[29] Rosentritt M, Ries S, Kolbeck C, Westphal M, Richter EJ, Handel G (2009). Fracture characteristics of anterior resin-bonded zirconia-fixed partial dentures. Clin Oral Investig.

[30] Malgaj T, Papšík R, Abram A, Kocjan A, Jevnikar P (2023). Bonding performance of surface-treated zirconia cantilevered resin-bonded fixed dental prostheses: in vitro evaluation and finite element analysis. Materials (Basel).

